# Exploring the reality of exosomes in dermatology^☆^

**DOI:** 10.1016/j.abd.2024.09.002

**Published:** 2024-11-18

**Authors:** Taciana Dal’Forno-Dini, Martina Souilljee Birck, Marco Rocha, Edileia Bagatin

**Affiliations:** a Private Practice, Porto Alegre, RS, Brazil; b Department of Dermatology, Hospital Santa Casa de Porto Alegre, Porto Alegre, RS, Brazil; c Department of Dermatology, Universidade Federal de São Paulo, São Paulo, SP, Brazil

**Keywords:** Exosomes, Extracellular vesicles, Dermatology

## Abstract

Exosomes are extracellular nanovesicles secreted by several cells in the human and animal body. Consisting of a lipid membrane and encapsulated proteins, they contain biologically active substances such as proteins, DNA, RNA, transcription factors, and metabolites. Discovered in the 1980s, exosomes play an important role in cell-to-cell communication and immune function. They vary in size, content, and function depending on the cell of origin. Exosomes have attracted interest in the field of Dermatology due to their potential applications in the treatment of scars, skin rejuvenation, hair regeneration, and other dermatological conditions. However, further clinical studies are needed to prove their efficacy and safety. Regulatory issues also need to be considered, as the use of exosomes in cosmetics and medical treatments is not yet fully approved in some countries. Moreover, it is important to understand the risks and side effects associated with the use of exosomes before their clinical use. Although promising, more research is needed to explore the full potential of exosomes in Medicine and Dermatology.

## Introduction

Exosomes are nanosized extracellular vesicles, approximately 30–150 nm in size,^1^, ^2^, ^3^, ^4^ derived from the endosomal pathway and secreted by several cell types, including stem cells, keratinocytes, fibroblasts, human and animal immune cells, and also by plant cells.^5^ They consist of a lipid bilayer replete with encapsulated surface proteins, isolating a biologically active cargo, including proteins, DNA, messenger RNA, microRNA (miRNA), transcription factors, membrane trafficking proteins, antigen-presenting proteins, other peptides, metabolites, and lipids.^3^, ^4^

They were first reported in 1983 by Johnstone et al. and initially called “cellular garbage carriers”.^3^, ^6^ In the 1990s, it was discovered that exosomes play a vital role in intercellular communication and immune function, as they express characteristics of the cell of origin, acting as paracrine molecules that interact with the extracellular matrix (ECM) and adjacent cells.^3^

Exosomes obtained from the same cell type can be highly variable based on the characteristics of the cell and its microenvironment, with different sizes, contents and functions in target or recipient cells.^4^, ^7^ Given their differences, exosomes are very heterogeneous in structure and function.^4^ Thus, their effects on recipient cells depend on their content and the different receptors on the cell surface.^1^

In recent years, exosomes have emerged as a promising treatment for the delivery of nucleic acid-based therapeutics due to their inherent biocompatibility, ability to cross physiological barriers and low immunogenicity. Because they are naturally generated by cells in the human body, they are believed to result in reduced inflammatory responses.^5^ Moreover, as an alternative to the use of regenerative cell therapy (the use of living cells, such as stem cells, to repair or replace damaged tissues), exosomes would theoretically have several advantages, including stability, low toxicity, biocompatibility, and reduced immune rejection and risk of tumorigenesis, as their use does not involve living cells.^8^

This review aims to independently address the recent great interest in the use of exosomes in Dermatology, based on the most recent published articles and research, providing a comprehensive summary of their real role in the healing process, scar treatment, skin rejuvenation, hair regeneration, skin neoplasms, inflammatory diseases, autoimmune diseases, acne, melasma, and other hyperpigmentation disorders, as well as discussing biosafety, regulatory issues, products available in the Brazilian market, irregular use, and future prospects. It is expected, therefore, to provide a greater understanding of the action mechanisms and possible current and future clinical applications of exosomes in Dermatology.

## Origin

Studies show that exosomes can be obtained from cells, tissues, or body fluids of almost all mammalian species. These sources include stem cells, cancer cells, immune cells, hair follicle stem cells (HFSCs), and human dermal fibroblasts (HDFs); saliva, blood, urine^8^ and platelets are also considered sources for exosome isolation.^1^ They demonstrate molecular transport capacity with biocompatibility, making them promising agents for intercellular and interspecies communication.^6^ Mesenchymal stem cells (MSCs) are pluripotent stem cells that originate from adult stem cells and have the capacity for self-renewal and self-repair, as well as the ability to differentiate into multiple functional cells under certain conditions.^4^ MSCs can be obtained from several sources, including adipose tissue derived stem cells (ADSCs), placental mesenchymal stromal cells (PMSCs), umbilical cord mesenchymal stem cells (UCMSCs), and bone marrow stem cells (BMSCs). Exosomes obtained from these cells are called ADSC-exos, PMSC-exos, UCMSC-exos, and BMSC-exos.^8^

Plant-derived exosomes are called plant-derived exosome-like nanoparticles (PELNs) and represent an attractive alternative due to their theoretical biosafety. PELNs have been reported to have regenerative, anti-inflammatory, and low immunogenic properties.^3^ In a recent literature review, PELNs were evaluated as promising substitutes for human exosomes due to their similarity in structure and function and were described as potential therapeutic agents for diseases of the digestive, respiratory, neurological, vascular, genitourinary, endocrine, and musculoskeletal systems. Their origin includes several sources, such as flowers, ginger, grapes, broccoli, asparagus, curcuma, lemon, grapefruit, garlic, soybeans, mushrooms, tomatoes, pears, and strawberries.^6^, ^9^

Some *in vitro* and animal studies have also evaluated the effects of exosome-like nanovesicles from *Phellinus linteus*, a medicinal fungus known for its antitumor and anti-inflammatory properties, and *Lactobacillus plantarum*, a bacterium capable of performing lactic fermentation.^5^, ^10^ Other exosomes derived from bacteria found in the human intestinal microbiota have also been reported.^11^

## Exosome isolation technique

The potential theoretical benefits of using exosomes in clinical practice also depend on the standardization of isolation methods. Techniques derived from ultracentrifugation are currently considered the reference method. However, other methods have been developed and include isolation by ultrafiltration, immunoaffinity and precipitation. The difficulty of these techniques lies in the exclusive isolation of exosomes, without the presence of other ECM components. The combination of techniques is described to ensure the isolation of pure exosomes in the sample; however, it increases costs and the need for more advanced technical training.^4^

After isolation, due to the variation in the final yield of exosomes, an analysis is necessary to characterize their purity and concentration. Electron microscopy provides morphological information, allowing the observation of structures bound to the exosome membrane. Techniques such as dynamic light scattering and nanoparticle tracking measure their size variation and concentration. Immunological methods, including flow cytometry and western blot, use specific markers to detect exosomes, which commonly express tetraspanins (CD9, CD63, CD81) and heat shock proteins (HSP70, HSP90). Mass spectrometry allows the identification and quantification of their molecular components, such as proteins, nucleic acids and lipids, and the quantitative reverse transcription polymerase chain reaction confirms and quantifies the RNA of exosomes.^4^, ^5^, ^8^

## Functions

Given the ability of exosomes to mediate cellular communication and the potential therapeutic applications based on their composition, researchers have begun to explore their role in Medicine, as part of a still unrecognized area called regenerative Medicine.^5^ The source from which exosomes are isolated is of critical importance regarding their functions and, therefore, clinical use.

Studies in the last decade have discovered functions of exosomes in cell survival, proliferation, migration, differentiation and senescence, immunomodulation, angiogenesis, and wound healing, as well as potential biomarkers of neoplastic and autoimmune diseases.^11^

They have recently begun to be explored for use in Dermatology due to their positive effects on healing in *in vitro* studies and in murine models, through the promotion of cell migration, ECM reconstruction, and angiogenesis.^4^ In the context of skin diseases, the discovery that exosomes modulate communication between skin cells and their microenvironment is believed to possibly explain the complex pathogenesis of chronic inflammatory and autoimmune dermatoses and, therefore, research has targeted them as promising for diagnosis and treatment.^12^

In cosmiatry, several studies investigate exosomes for aesthetic purposes and cell regeneration, but most studies are still in the preclinical phase. Despite the plethora of studies developed in the last decade, few researchers have published promising preclinical results, with studies in humans.^4^ Therefore, more well-designed clinical studies, mainly randomized ones, controlled with a vehicle group, are still required to justify, with efficacy and safety, the use of exosomes and their variations.

## Exosomes in healing and scar treatment

Healing is a complex process that comprises distinct and overlapping phases.^13^ It is known that the presence of inflammation is crucial for skin regeneration. However, persistent inflammation is not beneficial, and the degree of inflammation has a significant impact on the entire process. The onset and disappearance of the inflammatory reaction are key to determining the quality and timing of the formation of regenerated tissue or scar. Studies show that, during the healing of skin wounds, stem cells have the ability to reduce inflammation, accelerate the proliferation phase, and help in tissue remodeling, acting through paracrine mechanisms, and releasing growth factors and exosomes.^8^

In recent years, exosomes have emerged as a promising treatment to improve wound healing due to their unique characteristics. Their diverse composition theoretically allows the regulation of multiple cell processes involved in wound healing, including inflammation, angiogenesis, cell proliferation, and ECM remodeling.^5^

### *In vitro* and animal studies

A systematic review published in 2022 found 105 preclinical studies on the use of exosomes in wound healing, of which 51 were performed in murine models. The most commonly used exosomes were those derived from humans (MSC-exos and UCMSC-exos), the most commonly used isolation technique was ultracentrifugation, and the most common mode of administration found in the studies was the subcutaneous injection. In this analysis, exosome therapy improved wound healing regardless of the mode and frequency of administration, concentration, or source.^14^

An animal model study showed that the intralesional injection of exosomes derived from mouse macrophages produced increased fibroblast proliferation and migration, collagen deposition, and endothelial cell stimulation, which reduced wound closure time.^15^

A meta-analysis on the use of exosomes in wound healing in diabetic mouse models showed a total of 21 studies with 323 animals. Therapy with exosomes of human stem cell origin, with many studies using ADSC-exos, was shown to be superior to control treatment in terms of wound healing rate, re-epithelialization, collagen deposition, and decrease in width after scar formation. Additionally, the expression of inflammatory factors was significantly lower with exosome treatment.^16^ A study showed that exosomes derived from menstrual blood increased angiogenesis and wound re-epithelialization in diabetic mice.^15^

Chen et al. evaluated the efficacy of the topical application of exosomes derived from human embryonic cells in pressure ulcers in aged mice, demonstrating faster wound closure and angiogenesis stimulation due to the probable rejuvenation of senescent endothelial cells.^13^

Regarding hypertrophic scars and keloids, ADSC-exos demonstrated ability to inhibit fibroblast production and activation in the ECM, preventing the emergence of abnormal scarring.^17^ In an animal model, combined therapies of HDF-exos associated with botulinum toxin type A and UCMSC-exos with fractional laser were more effective for hypertrophic scars when compared with the use of isolated treatments.^17^, ^18^ The combination of UCMSC-exos incorporated into hydrogel seems to more effectively prevent the formation of hypertrophic scars compared with exosomes injected as monotherapy, over a four-month follow-up period.^19^

Some preclinical studies and *in vitro* experiments with PELNs have yielded promising results, demonstrating the ability to deliver bioactive molecules, modulate cell responses, and improve tissue repair processes. In a study conducted by Sahin et al., it was observed that PELNs derived from wheat promote the production of type I collagen, as well as the proliferation and migration of fibroblasts and immune cells, with antiapoptotic activity in human fibroblasts and keratinocytes. Furthermore, it was found that these PELNs induce angiogenesis in human umbilical vein endothelial cells, demonstrating their potential use for tissue regeneration. Moreover, PELNs can enhance neurogenesis, which ensures an adequate supply of neuropeptides for the healing tissue, facilitating its regeneration by stimulating the neural differentiation of MSCs. *In vitro* studies with ginseng-derived exosomes have demonstrated their ability to stimulate neurogenesis in mammalian MSCs by transferring their miRNA, suggesting their use may enhance neural differentiation and stimulate wound healing.^20^

### Clinical studies

Regarding the healing process, a clinical trial with platelet-derived exosomes injected once into lesions generated by skin biopsy in 11 healthy patients demonstrated no difference in healing time compared to lesions that were not treated with exosomes.^21^

Kwon et al. published in 2020 a split-face clinical trial with 25 patients showing that the combination of ADSC-exos in drug delivery after fractional CO_2_ laser for the treatment of acne scars on the face, in three sessions every three weeks, produced less erythema at treatment sites, shorter post-treatment downtime (4.1 days vs. 4.3 days), and a significant improvement in the Acne Scar Assessment Scale and the Investigator's Global Assessment, at the discretion of the evaluators themselves for scars from the second session on, compared to treatment with CO_2_ laser alone (improvement of 32.5% vs. 19.9%, respectively). Adverse effects, such as pain and edema after treatment, were slightly lower on the side treated with ADSC-exos drug delivery, although not statistically significant.^22^

## Exosomes in skin rejuvenation

The effects of skin aging are one of the most common reasons for dermatological consultation. Internal and external factors contribute to skin aging, including ultraviolet radiation (UVR), pollution, and smoking. Photodamage, in particular, produces changes in the ECM, including a decrease in collagen and elastic fibers, leading to the clinical appearance of wrinkles and changes in skin firmness and texture. Due to their ability to modulate cell communication and fibroblast functions, exosomes have gained much attention in recent years for their potential therapeutic usefulness in skin rejuvenation.^23^

### *In vitro* and animal studies

Recent review articles have analyzed preclinical studies on the use of exosomes in the treatment of photoaging, concluding that, by reducing inflammatory markers and upregulating the ECM, it is possible to prevent fibroblast senescence by stimulating type I collagen, elastin, fibronectin production, and decreasing type III collagen expression.^3^, ^24^

A study showed that ADSC-exos, when injected into the photoaged skin of mice, produced a significant reduction in epidermal thickness and an increase in dermal thickness after seven days of treatment, as well as a reduction in skin wrinkles in mice with UVR-induced photoaging.^25^ *In vitro* experiments with these exosomes demonstrated potential stimulation of fibroblast proliferation and reduction of intracellular free radical production, indicating an associated antioxidant effect.^24^ *In vitro* studies with BMSC-exos in photoaged dermal fibroblasts showed a reduction in oxidative stress and apoptosis induced by UVB radiation. Pretreatment with UCMSC-exos (before sun exposure) and treatment with HDF-exos also showed a protective effect on dermal fibroblasts.^5^

Exosomes from the fungus *Phellinus linteus* and the bacterium *Lactobacillus plantarum* showed antioxidant and antiaging activity *in vitro*, by inducing fibroblast proliferation and regulating genes related to the ECM.^5^, ^10^, ^26^

### Clinical studies

Park et al. evaluated the clinical efficacy of ADSC-exos applied prior to microneedling with 1-mm needles to treat facial skin aging. In a prospective and randomized split-face study lasting 12 weeks, 28 patients underwent three sessions at a three-week interval. In each treatment, ADSC-exos were applied to one side of the face followed by microneedling, while saline solution and microneedling were applied to the other side as control. The Global Aesthetic Improvement Scale (GAIS) score was significantly higher on the exosome-treated side six weeks after the first session. At the end of 12 weeks, the participants who received the ADSC-exos treatment had a higher GAIS score (28% vs. 14%), as well as a greater reduction in wrinkles (12.4% vs. 6.6%), improved skin elasticity (11.3% improvement on the ADSC-exos-treated side vs. 3.3% worsening in the control group), improved hydration (6.5% vs. 4.5%) and pigmentation (9.9% vs. 1%).^27^ Another clinical trial involving 21 women treated for hyperpigmentation with the topical application of the same exosomes twice a day for eight weeks, showed a significant decrease in melanin levels, assessed with Mexameter®, four weeks after treatment as evaluated by indirect methods. However, the skin-clearing effect was reduced over the following four weeks.^28^

In a randomized, double-blind, placebo-controlled study, the topical application of PMSC-exos after microneedling showed good results in skin quality. The 20 patients who underwent treatment with exosomes in three monthly sessions showed apparent improvement in wrinkles, pores, oiliness, uniformity, and skin vascularization 120 days after the last session, with no description of the statistical analysis of these results. Satisfaction with exosome treatment, - was significantly higher compared to the placebo group and there were no reports of adverse effects.^29^ The topical application of the same exosomes (PMSC-exos) immediately before the injection of calcium hydroxyapatite into the face of five patients demonstrated, after 30 days, a faster clinical response (21 days vs. 30 days) in the evaluation of researchers and participants, when compared to injectable biostimulator in monotherapy.^30^

A report of two cases of topical application of stem cell-derived exosomes after a facial resurfacing procedure with a fractional ablative laser resulted in faster recovery, after about five days, compared to laser alone, which showed a re-epithelialization time of seven to ten days.^31^

Regarding the use of exosomes of non-human origin in rejuvenation, a Korean study performed the topical application of exosomes derived from *Lactobacillus plantarum*, at a dose of twice a day for four weeks, in the periorbital region of 16 women aged an average of 50 years, demonstrating, in image analysis systems, a reduction of 15.89% in wrinkles and 8.5% in pigmentation at the end of the follow-up period.^26^

## Exosomes in hair regeneration

Hair loss is one of the obvious phenotypes associated with aging. It is known that aging-associated alopecia is linked to follicular quiescence and hair miniaturization.^8^ In normal hair, growth occurs at the level of the hair follicle as a continuous three-phase cycle: anagen (active growth), catagen (transition and involution) and telogen (rest). Dermal papilla cells (DPCs), responsible for hair follicle development, are known to release several growth factors, communicating with epithelial cells, germ cells and stem cells. These, in turn, are responsible for normal hair growth and proliferation during a new hair cycle. Given the critical role that DPCs play in the hair follicle and hair cycle, they have been used as a source of exosomes, as they upregulate Wnt/β-catenin signaling, a key cellular pathway involved in hair regulation, regeneration and morphogenesis. Exosomes derived from ADSCs, BMSCs, keratinocytes and other MSCs have also been studied.^4^, ^6^, ^32^

### *In vitro* and animal studies

Preclinical evidence for the use of exosomes in the treatment of androgenetic alopecia (AGA) has mostly used DPCs-exos.^33^ In a murine model, intradermal injections of DPCs-exos induced hair growth compared to placebo by converting the hair follicle from the telogen to the anagen phase and delaying the transition from the anagen to the catagen phase observed both clinically and on histopathology.^4^, ^6^ DPCs-exos also reduced hair loss and perifollicular inflammation in mice with alopecia areata.^33^

A study in a murine model with AGA compared intradermal injection of MSC-exos associated with a formula containing medicinal herbs traditionally used in China for the treatment of AGA, with topical minoxidil and the same formula as control, demonstrating earlier hair regeneration than in controls, through the conversion of hair follicles from the telogen to the anagen phase, observed clinically and on histopathology.^34^

*In vitro* and animal research with ADSC-exos demonstrated stimulation of the proliferation, migration and induction of DPCs, promotion of hair shaft elongation and hair growth, and a neutralizing action on the effects of dihydrotestosterone, ensuring an increase in the number of follicles and hair thickness.^35^ An *in vivo* study in mice demonstrated greater clinical and histopathological efficacy of hair transplantation when the graft contained ADSC-exos, in addition to dermal and epidermal cells, with significant hair regeneration.^36^

Plant-derived exosomes or PELNs also seem to promote hair growth through activation of the Wnt/β-catenin pathway and release of growth factors. A study in a murine model evaluated the efficacy of garlic-derived exosomes administered orally, at two different doses, and topically in hair regeneration. Mice that received exosomes orally at a high dose demonstrated increased the Wnt-B-catenin pathway signaling and hair follicles with larger diameters. The low-dose oral route produced similar effects, clinically and on histopathology, to the topical use.^37^

### Clinical studies

In a case series, 39 patients with mild to moderate AGA received human ADSC-exosomes applied topically after microneedling, in weekly sessions for 12 weeks, demonstrating a significant increase in hair density (increase of 24.9 hairs/cm^2^) and thickness (increase of 8.8 μm) at the end of the follow-up.^38^

A case report of a 38-year-old male patient with AGA and poliosis demonstrated apparent hair growth and repigmentation of white hair after four monthly sessions of 1064 nm Nd:YAG picosecond fractional laser followed by drug delivery of exosomes derived from the *Rosa x damascena*.^9^

## Exosomes in skin neoplasms

### Melanoma

Melanoma is responsible for 80% of deaths from skin neoplasia. With its incidence increasing in recent years, early diagnosis and detection of biomarkers, especially those associated with prognosis, monitoring of disease progression, and response to treatment, are necessary. Thus, exosomes have played a significant role in cutaneous oncology due to their capacity for immune evasion and, thus, inducing the formation of a pre-metastatic niche in several types of cancer. Exosomes derived from cells of melanoma patients are potential “liquid biopsies” and, because they carry the genetic load of their cell of origin, are promising tools for diagnosis and prognosis.^39^ Currently, researchers are working to determine the metabolic profile and exosomal signature specific for melanoma; however, the available data are limited to animal models and *in vitro* cells.^40^

### Squamous cell carcinoma

Preclinical evidence suggests that exosomes are linked to the pathogenesis of squamous cell carcinoma by enhancing the activity of proteins involved in cell translation, transcription, and cell division signaling of mutated keratinocytes. However, further studies are required to understand their mechanisms and potential clinical use.^41^

### Mycosis fungoides

Although the role played by exosomes in the most common form of cutaneous lymphoma is still poorly understood, given the overexpression of certain miRNAs from exosomes of patients with mycosis fungoides, especially miR-155 and miR-1246, they have been studied as potential diagnostic and prognostic biomarkers and as targets for new therapies.^42^, ^43^

## Exosomes in autoimmune and inflammatory diseases

### Psoriasis

The pathophysiology of psoriasis continues to be studied and, in recent years, many discoveries have been made. *In vitro* studies with exosomes from patients with psoriasis demonstrate potent activation of the inflammatory cascade and increased oxidative stress due to their high power of intercellular communication and relationship with the immune system.^44^ Exosomes from patients treated with secukinumab, adalimumab, and ustekinumab, when compared with untreated patients, demonstrated differences in their lipid composition, with a qualitatively higher lipid load in patients using immunobiologicals, which emphasizes the reduction in the cardiovascular risk when the disease is controlled, and the role of exosomes in the therapeutic follow-up.^45^

*In vitro* and murine model studies have shown that the injection of UCMSC-exos reduces epidermal proliferation, disease extension, and severity, and that the topical use of exosomes derived from human embryos and ADSC reduces the levels of interleukins, such as IL-17.^44^, ^46^, ^47^, ^48^ More recently, the use of exosomes as a means of topical delivery of tofacitinib increased its therapeutic effects and reduced adverse events in mice.^48^

### Atopic dermatitis (AD)

The ability to modulate the immune system with the use of exosomes makes them agents likely to be involved in the pathogenesis of AD, but also possible therapeutic agents.^49^

*In vitro*, HDF-exos promoted increased barrier function and skin recovery in chronically inflamed cells^49^, ^50^ and ADSC-exos reduced inflammatory pathways and angiogenesis. In mice with AD, when administered subcutaneously and intravenously, these same exosomes promoted a reduction in serum IgE levels and symptom attenuation.^41^

A clinical trial investigating the efficacy of a single dose of human UCMSC-exos applied subcutaneously in 34 atopic adults demonstrated improvement in disease activity and severity scores, with no adverse events. The intravenous use of allogeneic BMSC-exos in adult patients with moderate to severe and refractory AD demonstrated significant symptom improvement in four of five patients for at least 38 weeks, with no adverse events.^51^

### Vitiligo

As in other autoimmune diseases, exosomes, due to their relationship with the immune system, are involved in the pathophysiology of vitiligo. *In vitro*, it was discovered that exosomes from patients with vitiligo are capable of inhibiting melanogenesis and reducing tyrosinase activity due to the presence of antibodies against melanocytes.^52^ However, the mechanism of action remains poorly understood and new studies are being developed to identify and use exosomes as disease biomarkers or even as a therapeutic option.^53^

## Exosomes in connective tissue diseases

### Systemic lupus erythematosus (SLE)

For the same reasons mentioned above, exosomes seem to be linked to the pathogenesis of SLE. Biomarkers associated with exosomes have been studied for the diagnosis and prognosis of the disease, especially in lupus nephritis, as well as possible targets for new therapies.^41^, ^42^, ^54^

### Scleroderma

Fibrosis caused by the exacerbated production of connective tissue due to dysfunction of dermal fibroblasts is at the heart of the pathophysiology of scleroderma. Studies with exosomes from scleroderma fibroblasts have demonstrated increased production and dysregulation of collagen I and fibronectin in normal fibroblasts.^41^, ^54^

As a therapeutic measure, the single application of UCMSC-exos in mice demonstrated inhibition of fibroblast proliferation, migration, and activation. Since skin ulcers are frequent complications of the disease, the use of exosomes for this purpose has been studied and seems to be promising.^52^, ^54^

### Dermatomyositis

*In vitro* studies demonstrate the excessive circulation of exosomes containing genetic cargo that activates autophagy of cells and muscle antibodies in patients with the disease, and that there was a reduction in their levels after starting anti-rheumatic therapy. Moreover, these exosomes seem to be linked to disease activity, presence of interstitial pulmonary disease, and vascular alterations.^52^

## Other applications

### Drug reactions

Exosomes from patients with severe drug reactions, such as Stevens-Johnson syndrome (SJS) and toxic epidermal necrolysis (TEN), have demonstrated the presence of a gene capable of overexpressing inflammatory pathways and promoting cell apoptosis, and the higher its concentration, the larger the area of ​​ skin affected. Patients with drug reactions with eosinophilia and systemic symptoms (DRESS) and generalized pustular psoriasis also have pro-inflammatory miRNAs in their exosomes, with activation of T cells.^52^

### Chronic cutaneous graft-versus-host disease (GVHD)

There is a case report in the literature of allogeneic bone marrow transplantation, which developed into uncontrolled GVHD and was treated with extracorporeal photopheresis, tacrolimus, imatinib, cyclosporine and high doses of corticosteroids. The case showed skin lesions such as papules, plaques, eczema, erosions and ulcerations, in addition to nail involvement and cicatricial alopecia, starting one year after the transplant. The intravenous use of PMSC-exos, in four sessions with a weekly interval, demonstrated improvement in skin involvement and laboratory profile 15 days after the last session, with no adverse events, intolerance or the emergence of infections during the follow-up period (five months). There is, however, no explanation as how this improvement occurred.^55^

## Biosafety

Exosome therapy is considered by many authors as a cell-free method; therefore, a therapy correlated with lower risks of tumorigenicity and immunogenicity, and reduced potential for uncontrolled cell differentiation and cell proliferation when compared to stem cell therapy.^7^ However, proteins, metabolites and nucleic acids delivered by exosomes to recipient cells effectively alter their biological response, potentially promoting or inhibiting diseases.^11^

Exosomes can, for example, limit or promote viral infections. Exosomal cargoes can suppress infection by limiting viral replication or increasing antiviral immunity, but they can also promote viral replication after hijacking the exosome biogenesis machinery. These can serve as a pseudoenvelope that improves viral entry, increasing its infectivity. The similarities in size, density, molecular charge, and use of common components to harness cell proteins and vesicle trafficking machinery between enveloped retroviruses, particularly HIV 1 and 2 and exosomes support this idea. Similarly, exosomes originating from immune cells and neoplastic cells release cargoes that can influence the immune system activity in the target cell, stimulating or suppressing its proliferation and function. Exogenous doses of supraphysiological levels of exosomes in mice have been associated with neoplastic induction and progression.^11^

Only a few clinical studies with allogeneic exosomes, in their methods, stated that infectious screening of the donor and the final product was performed, which included blood typing and Rh factor, research on viral diseases, and sexually transmitted infections.^22^, ^23^, ^29^, ^30^

Furthermore, there is no consensus/standardization or current regulation on safe extraction, purification, quantification, concentration methods, dose, and posology, which makes it difficult to conduct multicenter clinical trials with a sufficient sample of patients.^23^

Regarding plant-derived exosomes, more studies are needed to ensure their safe application. A comprehensive understanding of their toxicological aspects, biodegradability and clearance dynamics is necessary.

## Regulatory issues

In Europe and the United States, the use of cosmetics based on products derived from humans is prohibited, as there is the possibility of transmission of viral or prion diseases. In December 2019, the FDA issued a safety notice regarding the use of exosomes following multiple reports of unspecified serious adverse events in patients in Nebraska who were treated with unapproved exosome-containing products.^56^

Although several *in vitro*, animal, and basic clinical studies have described the potential benefits of exosomes, the FDA has not yet approved exosomes for use as topical, injectable, or intravenous treatments. However, in the United States, six companies produce and supply products with exosomes for clinical use, all of which are of human origin, without specifying their source.^3^

In Brazil, the National Health Surveillance Agency (ANVISA, *Agência Nacional de Vigilância Sanitária*) authorizes the use of exosomes of non-human origin in the group of grade 2 cosmetics, which means that the products must be for external use, that is, they can only be applied to the skin, with an intact epidermis, therefore not allowing their use by injection or as drug delivery. The presentation in sterile vials, however, encourages their use by injection or as “drug delivery” after procedures that cause disruption of the skin barrier, such as microneedling or fractional lasers.^23^, ^57^

## Available products in Brazil

The combination of allogeneic exosomes derived mainly from adult human stem cells, with over-the-counter moisturizers and serums is a growing trend in the global market. The quantity, purity and safety of the exosomes in these products are undefined since there is no regulation or post-marketing surveillance.^23^

Up until this revision, In Brazil, the products registered by ANVISA as cosmetics include Inno-Exoma®Exo-skin by INNOAESTHETICS, marketed by Suprema Marcas, and ASCE plus SRLV (for the skin), HLRV (for hair) and ILRV (intimate products) developed by BENEV Inc. in partnership with ExoCoBio.

The commercial product Inno-Exoma® Exo-skin was developed using NARBEX (Non-Animal Regenerative Bioengineering Exosome) technology which utilizes bioengineering to mimic cell-derived exosomes. Since it is considered a bioidentical product the company -calls it exosome-like or synthetic exosomes. It is presented in a sterile vial, and each box contains a 10 mL vial of lyophilized exosomes, associated with a complex of amino acids and peptides, mannitol and hyaluronic acid, accompanied by a 2 mL vial of saline solution for dilution. There are, as of now, no published clinical studies on this product.

ASCE Plus products are exosomes derived from the *Rosa damascena* plant, unlike the exosomes derived from mesenchymal cells marketed by ExoCoBio in other countries, such as South Korea. It is presented in a sterile vial, and the box sold in Brazil contains a 20 mL vial of exosomes, containing a complex of amino acids and peptides and other active ingredients such as ascorbic acid, retinol, nicotinamide and thiamine, as well as a 5 mL vial as diluent, which contains water, sodium bicarbonate, sodium chloride, sodium hyaluronate and an amino acid complex. Its use was evaluated in a single case report in the treatment of AGA and poliosis after four monthly sessions of fractional picosecond laser and drug delivery of the product, demonstrating hair growth and repigmentation of white hair, without adverse effects.^9^ There are no other published studies on this product.

Two systematic reviews published in 2024, included nine studies in Dermatology-: tissue regeneration (1), AD (2), melasma (1), rejuvenation (1), sensitive skin (1), melanin synthesis (1), skin glow (1), and as adjuvant to laser for acne scars (1). The authors highlight the large number of publications (*in vitro* studies, cell cultures, animal models,)as reviews on perspectives of possible exossomes aesthetic benefits.^56^, ^58^ However, there is a lack of clinical studies with good methodology and long-term follow-up. Besides, The presence of numerous components in the vials and the associated use of techniques, even minimally invasive ones, such as fractional laser and microneedling, prevent conclusions about the actual benefit of exosomes. There are no conflicts of interest between the authors and any company involved in the commercialization of exosomes and derivatives.

## Conclusion

Despite the infinite possibilities for future applications, there are important issues regarding exosomes that need to be elucidated before their use is authorized: 1) Identification of ideal cell sources for specific conditions/diseases; optimization of isolation methods and their characterization; 2) Standardization of large-scale production in compliance with current good manufacturing practice regulations and consideration of regulatory factors; 3) Establishment of dosing regimens (the appropriate amount, but also the required administration frequency); 4) Identification of the most efficient route of administration; 5) Understanding of the biodistribution and elimination of exosomes *in vivo*; 6) Overcoming the limitations caused by the lack of placebo-controlled studies; 7) Careful assessment of long-term risks and toxicity. All of these considerations are very important, especially since most of these studies involve the use of genetic cargo derived from human stem cells, without having yet established their true efficacy and safety.

Medical doctors and patients appreciate innovation, but they also place great value on safety. Despite the promising preclinical data, it is difficult to extrapolate findings in cell cultures and animal models to the complexity of human physiology. Further studies are needed to understand all the issues related to the use and applicability of exosomes in Medicine and Dermatology. Determining the potential risks and side effects associated with the use of exosomes is essential for their approval and starting their clinical use.

## Financial support

None declared.

## Authors’ contributions

Taciana Dal’Forno-Dini: Design and planning of the study; collection of data, or analysis and interpretation of data; drafting and editing of the manuscript or critical review of important intellectual content; collection, analysis and interpretation of data; effective participation in research orientation; critical review of the literature; approval of the final version of the manuscript.

Martina Souilljee Birck: Design and planning of the study; collection of data, or analysis and interpretation of data; drafting and editing of the manuscript or critical review of important intellectual content; collection, analysis and interpretation of data; critical review of the literature; approval of the final version of the manuscript.

Marco Rocha: Design and planning of the study; collection of data, or analysis and interpretation of data; drafting and editing of the manuscript or critical review of important intellectual content; collection, analysis and interpretation of data; effective participation in research orientation; critical review of the literature; approval of the final version of the manuscript.

Edileia Bagatin: Drafting and editing of the manuscript or critical review of important intellectual content; effective participation in research orientation; critical review of the literature; approval of the final version of the manuscript.

All authors approved the final version of this manuscript.

## Conflicts of interest

None declared.
